# Lipid Bilayers Are Long-Lived on Solvent Cleaned Plasma-Oxidized poly(dimethyl)siloxane (ox-PDMS)

**DOI:** 10.1371/journal.pone.0169487

**Published:** 2017-01-04

**Authors:** K. M. Rifat Faysal, June S. Park, Jonny Nguyen, Luis Garcia, Anand Bala Subramaniam

**Affiliations:** 1 Department of Physics, School of Natural Sciences, University of California Merced, Merced, CA, United States of America; 2 Department of Bioengineering, School of Engineering, University of California, Merced, Merced, CA, United States of America; Oregon State University, UNITED STATES

## Abstract

Although it is well known that phospholipids self-assemble on hydrophilic plasma-oxidized PMDS surfaces (ox-PDMS) to form cell membrane mimetic bilayers, the temporal stability of phospholipid membranes on these surfaces is unknown. Here we report that phospholipid bilayers remain stable on solvent-cleaned ox-PDMS for at least 132 hours after preparation. Absent solvent cleaning, the bilayers were stable for only 36 hours. We characterized the phospholipid bilayers, i) through quantitative comparative analysis of the fluorescence intensity of phospholipid bilayers on ox-PDMS and phospholipid monolayers on native PDMS and, ii) through measurements of the diffusive mobility of the lipids through fluorescence recovery after photobleaching (FRAP). The fluorescence intensity of the phospholipid layer remained consistent with that of a bilayer for 132 hours. The evolution of the diffusive mobility of the phospholipids in the bilayer on ox-PDMS over time was similar to lipids in control bilayers prepared on glass surfaces. Solvent cleaning was essential for the long-term stability of the bilayers on ox-PDMS. Without cleaning in acetone and isopropanol, phospholipid bilayers prepared on ox-PDMS surfaces peeled off in large patches within 36 hours. Importantly, we find that phospholipid bilayers supported on solvent-cleaned ox-PDMS were indistinguishable from phospholipid bilayers supported on glass for at least 36 hours after preparation. Our results provide a link between the two common surfaces used to prepare *in vitro* biomimetic phospholipid membranes—i) glass surfaces used predominantly in fundamental biophysical experiments, for which there is abundant physicochemical information, with ii) ox-PDMS, the dominant material used in practical, applications-oriented systems to build micro-devices, topographically-patterned surfaces, and biosensors where there is a dearth of information.

## Introduction

Nanometric phospholipid bilayers supported on a solid surface are cell membrane mimetic *in vitro* constructs useful for fabricating biosensors[1–6] for studying membrane biophysics[7–11], and for studying the binding of drugs[12]. Although glass (silicon dioxide) surfaces are used conventionally to support phospholipid bilayers[13], oxidized poly(dimethyl)siloxane (PDMS) surfaces—due to its ability to be molded into complex topographies[11,14,15], to be formed into microfluidic channels[8,16–18], and to be mechanically deformed[19]—are attractive substrates to support phospholipid bilayers[15,20–22]. Native PDMS is hydrophobic[23–28], plasma oxidation is required to make the surface of PDMS hydrophilic and thus suitable for supporting phospholipid bilayers. Over time however, the surface of oxidized-PDMS (ox-PDMS) regains its hydrophobicity in a process called “hydrophobic recovery”[23–28]. Recovery is favored thermodynamically, since native PDMS has a lower surface free energy than ox-PDMS. Conditions of storage, however, determine the rate (i.e. the kinetics) of hydrophobic recovery: ox-PDMS stored in ambient atmosphere becomes hydrophobic rapidly, while immersion in aqueous environments, certain chemical treatments[25], including adsorption with biomolecules[29], decreases and sometimes completely inhibits hydrophobic recovery[23–28]. Although PDMS is used widely in soft-lithography, the temporal stability of phospholipid bilayers on plasma-oxidized PDMS (ox-PDMS) is not known. Such a study is useful, particularly since the use of ox-PDMS as a biointerface, instead of glass, is advantageous in applications such as microfluidics[6], applications that require variations in topography and curvature[8,10,11,14,15,30], and applications that require dynamic changes in surface area[14,19].

In this paper, we study the temporal stability of model 1,2-dioleoyl-sn-glycero-3-phosphocholine (DOPC) phospholipid layers on ox-PDMS surfaces. As controls, we also prepared lipid monolayers on native PDMS and lipid bilayers on glass substrates. We quantified the characteristics of the phospholipid layers on ox-PDMS and the control surfaces over the course of 132 hours, i) by monitoring the evolution of the fluorescence intensity of the layers obtained through high-resolution laser scanning confocal microscopy imaging, and ii) by measuring the diffusive mobility of the phospholipids in the layers through fluorescence recovery after photobleaching (FRAP). The fusion of fluorescently-labeled small unilamellar vesicles (SUVs) on these three substrates produced phospholipid layers with qualitatively uniform fluorescence intensity. Quantitative analysis revealed that the pixel intensities were normally distributed for both the lipid monolayers on native PDMS and the lipid bilayers on both glass and ox-PDMS. The fluorescence intensity of phospholipid bilayers on ox-PDMS and phospholipid monolayers on native PDMS were clearly distinguishable from each other: bilayers exhibited twice the mean intensity of monolayers, and the distribution of pixel intensities of monolayers and bilayers did not overlap significantly up to ~ 2.5 standard deviations from their respective mean values.

The measured fluorescence intensity of the lipid layers formed on ox-PDMS was consistent with the interpretation that the lipid bilayer remained stable on the surface for the duration of the 132-hour experiment. Diffusion measurements, a second line of evidence independent from measurements of fluorescence intensity, further supported our observations: the diffusive mobility of lipids (evaluated through calculations of the diffusion coefficient from FRAP recovery curves) on ox-PDMS was similar to the diffusive mobility of lipids on glass [13,31]. Although the bilayers on ox-PDMS developed a mottled appearance 36 hours after preparation, our analysis revealed that the pixel intensities remained consistent with those of lipid bilayers.

The stability of the bilayer on ox-PDMS was highly dependent on cleaning prior to deposition of the bilayer. Without a three-step sequential sonic cleaning in acetone, isopropanol, and water prior to plasma oxidation, lipid bilayers on ox-PDMS degraded after 36 hours. Large patches of lipids peeled off the surface. In contrast, the properties of phospholipid bilayers supported on ox-PDMS that was cleaned with the three-step sonication procedure were indistinguishable, other than the mottled appearance, from the properties of phospholipid bilayers supported on glass surfaces up to 132 hours after preparation. Our results demonstrate that ox-PDMS may be used interchangeably with glass for applications requiring short and medium term stability of supported-lipid bilayers.

## Materials and Methods

### Materials

Sylgard 184 poly(dimethyl)siloxane silicone elastomer base and silicone elastomer curing agent (Dow Corning, 0.5 kg kit) was purchased from Krayden, Inc. (Denver, CO). Polystyrene Petri dishes (135 mm diameter), acetone (certified ACS grade), isopropanol (laboratory grade), glass slides (Corning Inc.) were all purchased from Fisher Scientific (Pittsburg, PA). Chloroform (anhydrous, ≥ 99% contains 0.5–1.0% ethanol as stabilizer), 1.0 M calcium chloride (CaCl_2_) solution (BioUltra, for molecular biology) were purchased from Sigma Aldrich (Missouri, MO). Ultrapure water (18.2 MΩ cm resistivity) was obtained from an ELGA Purelab Ultra water purification system (Woodridge, IL).1,2-dioleoyl-sn-glycero-3-phosphocholine (DOPC), 1,2-dioleoyl-sn-glycero-3-phosphoethanolamine-N-(lissamine rhodamine B sulfonyl) (ammonium salt) (Rh-DPPE) were all purchased from Avanti Polar Phospholipids (Alabaster, AL). Tris Buffered Saline (TBS) tablets (ultra pure grade) was purchased from VWR (Radnor, PA).

### Procedure for preparation of PDMS substrates

We mixed nine parts of elastomer to one part of curing agent and poured 6.8 g of the mixture into a 135 mm diameter Petri dish. After degassing in a vacuum chamber for 30 minutes (to remove trapped air bubbles) and curing for three hours in an oven set at 65° C, we obtained a PDMS disk that was 135 mm in diameter and 1 mm thick. To clean the surface, we sonicated the PDMS disk, sequentially, for a duration of 15 minutes each, in neat acetone and neat isopropanol. At the end of the procedure, the PDMS appeared slightly opaque due to the swelling of the elastomeric chains with the non-polar solvent molecules. The PDMS disk was rinsed with ultrapure water several times and then sonicated for a final 15 minutes in fresh ultrapure water before being placed overnight in an oven at 65° C. This step was to evaporate residual solvent and water—the PDMS regained its optical clarity. In one series of experiments, we omitted the cleaning steps to determine the effect of cleaning on the behavior of phospholipid layers on PDMS.

### Procedure for preparing SUVs

Small unilamellar vesicles (SUVs) were prepared using a previously disclosed method [14]. Briefly, we evaporated a given volume of phospholipid solution at a concentration of 10 mg/mL in a clean 4 mL glass vial (Wheaton) under a gentle stream of ultrapure nitrogen to obtain 4 mg of phospholipids as a dried film. We placed the vial in a vacuum chamber overnight to remove all traces of residual solvent. We rehydrated the film with 4 mL of Tris-HCl buffer at pH 7.5 with 2 mM CaCl_2_ for at least 15 minutes under vigorous manual agitation to dislodge the phospholipids from the wall to form multilamellar vesicles. We then sonicated the turbid suspension of multilamellar vesicles using a QSonic probe sonicator at an amplitude of 5% for 10 minutes. The vial was placed in an ice bath to limit heating. The solution turned translucent as the multilamellar vesicles broke into SUVs. We transferred the suspension of SUVs into 1.5 mL Eppendorf tubes and centrifuged the suspension for 10 minutes at 21.1 × G to pellet titanium particles that were dislodged from the sonicator tip. The supernatant, which contained the SUVs, was transferred to clean Eppendorf tubes and used within 1 week of preparation.

### Procedure for preparing self-assembled phospholipid layers on the substrates

We cut 12. 7 mm (½ inch) diameter disks of PDMS out of the larger pieces using biopsy punches and placed the substrates in the middle of a chamber made from a ring-shaped PDMS gasket 22 mm in diameter that was permanently bonded to a glass slide. The whole setup did not contain any glues or grease which might affect the properties of a supported bilayer.

Hydrophobic (native) PDMS surfaces were cleaned using the three-step sonication procedure and were used as is. We added 900 μL of the Tris-HCl buffer with 2 mM of CaCl_2_ and 45 μL of the SUV suspension into the chamber. After a 60-minute incubation period, we washed the surface of the PDMS by pipetting vigorously and exchanging the buffer in the chamber 10 times to remove excess unfused SUVs from the bulk. The glass cover slip and ox-PDMS were exposed to an air plasma in a Harrick Plasma PDC-002 plasma cleaner (high RF power) for 30 seconds before preparing the lipid layer according to the procedure described above.

### Conditions for imaging

We used a Zeiss LSM700 laser scanning confocal microscope mounted on an upright AxioObserver stand equipped with a W-Plan-Apochromat 63× water dipping objective with a numerical aperture of 1.0 to image all our samples. To conduct quantitative studies, we imaged all our samples identically: we excited our samples with a 555 nm diode laser at 1.0% power, and used a 585 nm longpass filter to collect the fluorescence emission of the rhodamine fluorophore. Each image was 50.7μm × 50.7 μm with a pixel size of 100 nm. We set the pinhole to 1.0 Airy Unit (corresponding to a slice thickness of 0.59 μm) and the PMT gain was set to 800. Images were captured at 12-bits which allowed intensity values to range from 0 to 4096 (arbitrary units).

For fluorescence recovery after photobleaching studies (FRAP), we chose imaging conditions that would lead to fast acquisition times to capture the dynamics of fluorescence recovery. We set our frame size to 148 × 148 pixels, and used a maximum rate of scanning with bidirectional acquisition. Each 20.2 μm × 20.2 μm image was acquired in 58.6 ms. Bleaching was carried out over a circular region with a nominal diameter of five μm with the 555 nm laser set at 100% laser power. The 555 nm laser was set at the lowest power (0.2%) to minimize acquisition photobleaching. The total time of 23.44 seconds for each FRAP experiment consisted of five images before the bleach pulse, a 10 millisecond bleach pulse, followed by 400 frames after the bleach pulse.

### FRAP analysis

We wrote a custom Matlab script to obtain the diffusion coefficients of the phospholipid layers from FRAP experiments[32]. The pixel intensities, *f(x)* along the diagonal (radial distance, *x*) of the first image post bleach was fitted with Eq 1 using the method of non-linear least squares to obtain the effective radius of the bleached area, *r*_*e*_ and the bleach depth, *K*.

$$f\left(x\right)=1-Kexp(-\frac{2x^{2}}{r_{e}^{2}})$$ Eq 1

A linear interpolation method was used to obtain the half-time of recovery, $\tau_{\frac{1}{2}}$ from the FRAP recovery curves. The half-time of recovery, effective radius of bleaching, and the nominal radius of bleaching, *r*_*n*,_ were then utilized to compute the diffusion coefficient (*D*) of the species (Eq 2).

D=rn2+re28τ12 Eq 2

The results that we obtained were similar to those obtained by fitting solutions of the diffusion equation to the recovery data[22] (data not shown).

## Results and Discussion

### Temporal evolution of fluorescence intensity of phospholipid layers on ox-PDMS, PDMS, and glass

**Fig 1:**
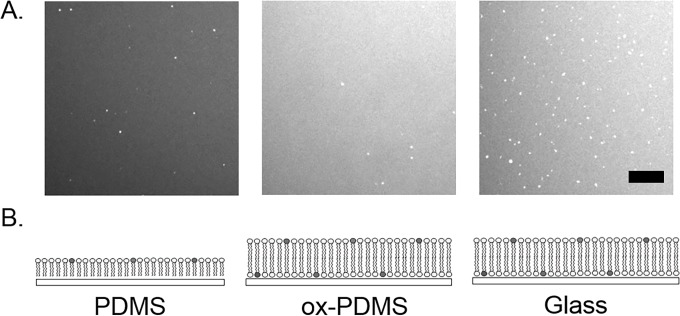
Representative confocal fluorescence images of lipid layers formed on PDMS, ox-PDMS, and glass immediately after preparation. A uniformly fluorescent lipid layer was present on all the surfaces after the fusion of SUVs. The layer on PDMS appeared to have a qualitatively lower intensity compared to the layers on ox-PDMS and glass. Unfused small unilamellar vesicles (SUV) appeared as bright compact spots on the lipid layers. The lower images are schematic diagrams of the configuration of the lipid molecules on the respective surfaces. Lipids form a monolayer on the PDMS, bilayers on ox-PDMS, and bilayers on glass. Scale bar 10 μm.

The phospholipid layers were prepared on ox-PDMS, PDMS, and glass through the fusion of small unilamellar vesicles (SUVs) composed of 99.9 mol % DOPC and 0.1mol % of the fluorescent phospholipid rhodamine-PE in Tris-HCl buffer containing 2 mM Ca^2+^. **Fig 1A** shows confocal fluorescence images of self-assembled phospholipid layers on, ox-PDMS, PDMS, and glass. On all surfaces, the fluorescent layer of adsorbed phospholipids appeared uniform. Small bright spots corresponded to adsorbed unfused SUVs. The phospholipid layer on PDMS appeared less bright than the layer on ox-PDMS and glass. The difference in intensity is consistent with the phospholipids forming a monolayer on the hydrophobic native PDMS and a bilayer on the hydrophilic ox-PDMS[21] (**Fig 1B**). It is known that fusion of SUVs produces phospholipid bilayers on hydrophilic glass surfaces[13,31].

**Fig 2:**
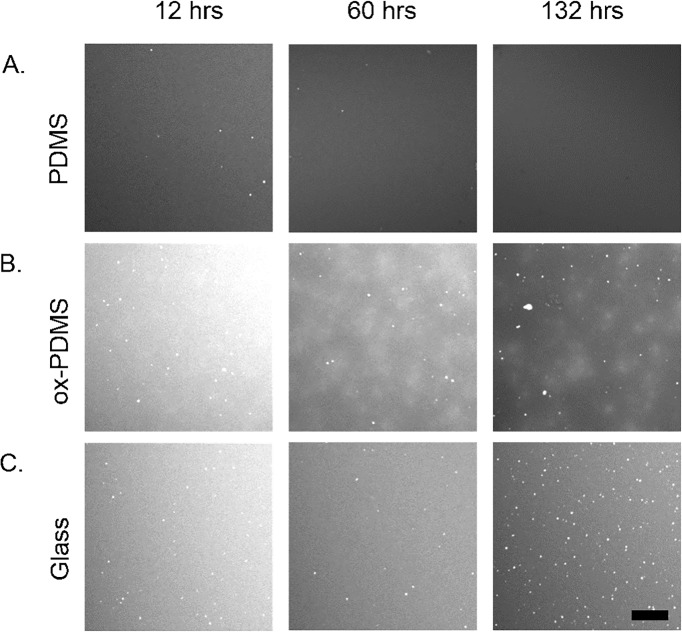
Representative confocal fluorescence images of the lipid layers on PDMS, ox-PDMS, and glass over time. Twelve hours after formation, all the lipid layers appeared similar to when they were first prepared. A) The monolayer on PDMS remained homogeneous and unchanged for 132 hours. B) The lipid bilayer on ox-PDMS appeared mottled at 60 hours. C) The homogeneity of the lipid bilayer on glass remains largely unchanged over the course of 132 hours. A large number of adsorbed SUVs form on the surface. Scale bar 10 μm.

We observed the three samples in parallel over the course of 132 hours. We imaged ten different locations on each of the surfaces to obtain representative snapshots of the evolution of the phospholipid layers. **Fig 2** shows representative images of the phospholipid layers, at 12 hours, 60 hours, and 132 hours. The phospholipid layers looked uniformly fluorescent on both native PDMS (**Fig 2A**) and glass (**Fig 2C**) over 132 hours, though the number of adsorbed small vesicles grew particularly numerous on the glass surfaces over time (**Fig 2C**). On ox-PDMS, the phospholipid layer, which was initially uniform, appeared mottled at 36 hours (**Fig 2B**). The mottled appearance persisted for 132 hours.

### Quantitative analysis of the fluorescence intensity of lipid layers

In principle, monitoring the evolution of lipid layers is straightforward—obtain the fluorescence intensity of the lipid layer over time and compare it with integer multiples of the intensity of lipid monolayers. Lipid monolayers have one lipid leaflet, whereas lipid bilayers have two lipid leaflets. Thus, the intensity of the lipid bilayer should be twice that of the lipid monolayer. In practice, since the fluorescence emission is from a relatively uniform molecularly thin film on a flat surface, even slight variations in the location of the surface with respect to the focal plane of the microscope will result in differences in apparent fluorescence intensity.

**Fig 3:**
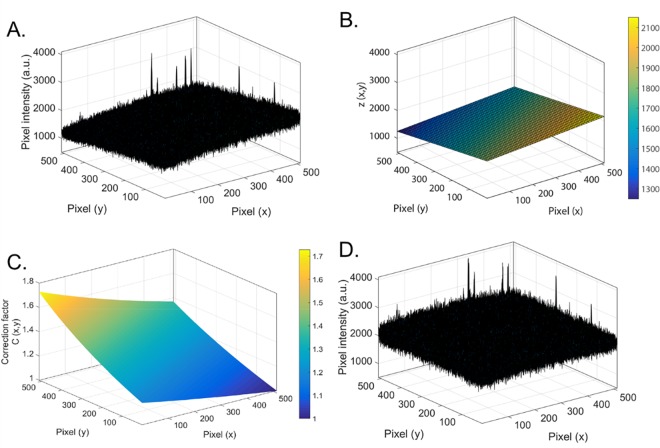
Correction for the effects of surface position on the values of intensity. A) Three dimensional representation of raw pixel intensities from a confocal image of a lipid bilayer on ox-PDMS. The x- and y-axis correspond to the pixel numbers while the z-axis corresponds to the intensity of the pixels. The image was 50.7 μm × 50.7 μm. It is clear that there are pixel-to pixel variations and a large-scale gradient that makes the plane of intensity values appear ‘tilted’. The tilt angle is relatively small ~ O*(0.01)* radians. Large peaks significantly above the mean fluctuation of the pixel intensities correspond to adsorbed SUVs. B) The result of a plane fit to the pixel intensities. The plane is a good representation of the position *z(x*,*y)* of the surface. C) A three-dimensional representation of the corresponding matrix of correction factors *C(x*,*y)* to account for the non-planar position of the substrate. D) The intensities after multiplication with the correction factors, demonstrating the removal of the large-scale gradient due to the varying position of the surface, while preserving the pixel-to-pixel fluctuations. S1 Fig.

We demonstrate this effect in **Fig 3A**, which is a three-dimensional plot of the pixel intensities of a confocal image of a lipid bilayer on ox-PDMS immediately after preparation. Along with pixel-to-pixel variations in intensity (and large peaks that corresponded to adsorbed SUVs), there is a gradient in the values of the intensity. Such gradients, which are present in all the images what we obtained (**S1 Fig**), is due to variations in the position of the surfaces within the focal plane.

Since we have a sub-resolution fluorescent layer on a non-fluorescent substrate, it is rational that the maximum intensity, after averaging out for pixel-to-pixel variations, corresponds to where the surface is in best-focus. Away from the region of best-focus, the intensity would decrease. With this in mind, we performed a non-linear least-squares fit of a plane to the intensities to determine the location of the surface *z(x*,*y)* (**Fig 3B**). We then obtained a matrix of correction factors, *C(x*,*y) = z*_*max*_/*z(x*,*y*,*)*, where *z*_*max*_ is the maximum value of *z* (**Fig 3C**). Multiplying *C(x*,*y)* with the raw intensity values corrects for the large-scale gradient while preserving pixel-to-pixel fluctuations in intensity (**Fig 3D**).

**Fig 4:**
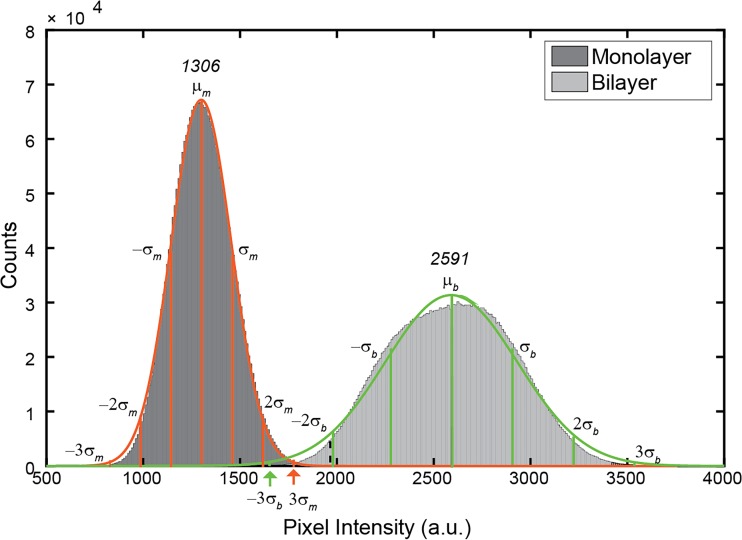
Histogram of pixel intensities of lipid bilayers on ox-PDMS and lipid monolayers on PDMS. Each histogram is obtained from *N* = 10 images, corresponding to a total count of ~ 2.6 × 10^6^ pixels per histogram. The orange continuous line is a fit of a normal distribution to the histogram for the monolayers. The green continuous line is a fit of a normal distribution to the histogram for the bilayers. The mean intensity of the monolayer, *μ*_*m*_ = 1360 is half the mean intensity of the bilayer, *μ*_*b*_ = 2591. The histogram of pixel intensities between the bilayers and monolayers do not overlap up to ~ 2.5σ from the mean. The clear separation of the distribution of pixel intensities allows classification of pixels as being monolayers or bilayers in images that potentially have mixed compositions of monolayers and bilayers.

**Fig 4** shows a histogram of pixel intensities from ten images of phospholipid bilayers on ox-PDMS and phospholipid monolayers on native PDMS immediately after preparation (each histogram consists of the intensity values of ~ 2.6 × 10^6^ pixels). Please see **S2 Fig** for the histogram from images not processed to remove gradients in intensity due to variations in the position of the surfaces. Despite the images appearing uniform qualitatively, the intensity of the pixels shows a clear distribution in values. The pixel intensities were well described by a normal distribution with a single mean, *μ*_,_ and standard deviation, *σ*. The mean intensity of the bilayer, *μ*_*b*,_ was, as expected, about 50 percent higher than the mean intensity of the monolayer, *μ*_*m*_. The distribution of intensities was also narrower for the monolayer, *σ*_*m*_ = 157 than the bilayer, *σ*_*b*_ = 312. The distribution of intensities was not limited to layers prepared on PDMS, pixel intensities of phospholipid bilayers prepared on glass surfaces were also normally distributed (**S3 Fig**).

The pixel intensities of monolayers were well-separated from those of bilayers. There is no overlap in the lower distribution of the intensities of bilayers up to– 2.3*σ*_*b*_ from *μ*_*b*_ and in the upper distribution of the intensities of the monolayer up to +2.4*σ*_*m*_ from *μ*_*m*_. This lack of overlap in intensities allows us to assign unambiguously pixels as being monolayers or bilayers. We assigned the region of overlap beyond 2.5*σ* as being indeterminate. **Table 1** lists the criteria that we used to assign identities to pixels based on their intensities. 1) Pixels were classified as bilayers when (*μ*_b_—2.3 σ_b_) < intensity < (μ_b_ + 2.3 σ_b_). 2) Pixels were classified as monolayers when (μ_*m*_− 5.1 σ_*m*_) < intensity < (μ_*m*_ + 2.4 σ_*m*_). 3) Pixels were classified as indeterminate when (μ_*m*_ + 2.4 σ_*m*_) < intensity < (μ_*b*_—2.3 σ_*b*_). Dark pixels (intensities < 500) were classified as holes, and bright pixels (intensities > 3308) were classified as adsorbed SUVs.

**Table 1:** Table of intensity values employed to classify pixels in lipid layers.

Classification	Intensity criteria	Intensity range (a.u.)
Hole	0 < Intensity < (μ_*m*_− 5.1 σ_*m*_)	0–500
Monolayer	(μ_*m*_− 5.1 σ_*m*_) < Intensity < (μ_*m*_ + 2.4 σ_*m*_)	500–1680
Indeterminate	(μ_*m*_ + 2.4 σ_*m*_) < Intensity < (μ_*b*_—2.3 σ_*b*_)	1680–1880
Bilayer	(μ_*b*_—2.3 σ_*b*_) < Intensity < (μ_*b*_ + 2.3 σ_*b*_)	1880–3308
SUV	Intensity > (μ_*b*_ + 2.3 σ_*b*_)	3308–4096

**Fig 5:**
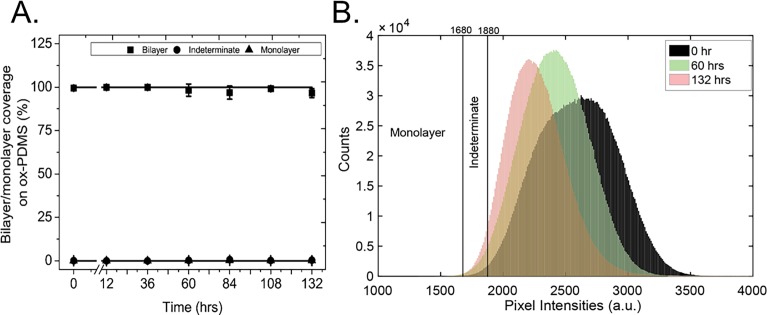
Quantification of the evolution of lipid bilayers on ox-PDMS as a function of time. A) The normalized area fraction of pixels with intensities consistent with lipid bilayers are represented by solid squares (■), the normalized area fraction of pixels with intensities consistent with lipid monolayers are represented by solid triangles (▲), and the normalized area fraction of pixels with indeterminate intensities are represented by solid circles (●). Standard deviations of the mean are represented by the corresponding error bars. The area fraction of bilayer was close to 100 percent for the duration of the 132-hour experiment. B) Plot of the histogram of pixel intensities (from *N* = 10 images at each time point) of lipid layers on ox-PDMS at 0 hours, 60 hours, and 132 hours after preparation. The peak of the distribution shifts with time signifying that the layer as a whole becomes less bright. The distributions show a longer right tail. The combination of movement of the peak of the distribution towards lower values and the development of a right tail should result in regions that are brighter when compared to the background, i.e. the mottled appearance of the layer. Note however, that the majority of pixels had intensities consistent with bilayers. The number of pixels classified as indeterminate increases slightly, and none of the pixels had intensities consistent with monolayers.

Using the classification listed in **Table 1**, we analyzed the ten images for each time point obtained at 24-hour intervals and normalized the results of our calculations by the total number of pixels per image to obtain an area fraction (**Fig 5A)**. The area fraction of phospholipid bilayer on the ox-PDMS surfaces was close to 100 percent for 132 hours. Hardly any pixels developed intensities consistent with monolayers. Thus, our analysis suggests that the phospholipid bilayers are stable on ox-PDMS surfaces for 132 hours.

To analyze our images further, we calculate and plot the histogram of pixel intensities of the lipid layer on ox-PDMS at 60 hours and 132 hours (**Fig 5B**). With aging, the peak of the distributions shifted towards lower values and the distributions exhibit a long right tail. A combination of the shift of the peak of the distribution of pixel intensities towards lower values, and the development of a right tail rationalizes the mottled appearance of the layer after 36 hours. Regions that are brighter (right tail values of the distribution) when compared to the background (the peak and lower values of the distribution) should lead to the appearance of bright and dark regions in the layer. Despite the shift in the distribution, the majority of pixels had intensities consistent with bilayers. The number of pixels classified as indeterminate increased slightly over time, but none of the pixels had intensities consistent with monolayers (**Fig 5B**).

### Fluorescence recovery after photobleaching (FRAP) measurements of phospholipid layers on PDMS

As a second independent means of characterizing the evolution of phospholipid bilayers on ox-PDMS, we measured the diffusive mobility of the phospholipids[33,34] in the lipid layers on ox-PDMS, PDMS, and glass using fluorescence recovery after photobleaching (FRAP).

**Fig 6:**
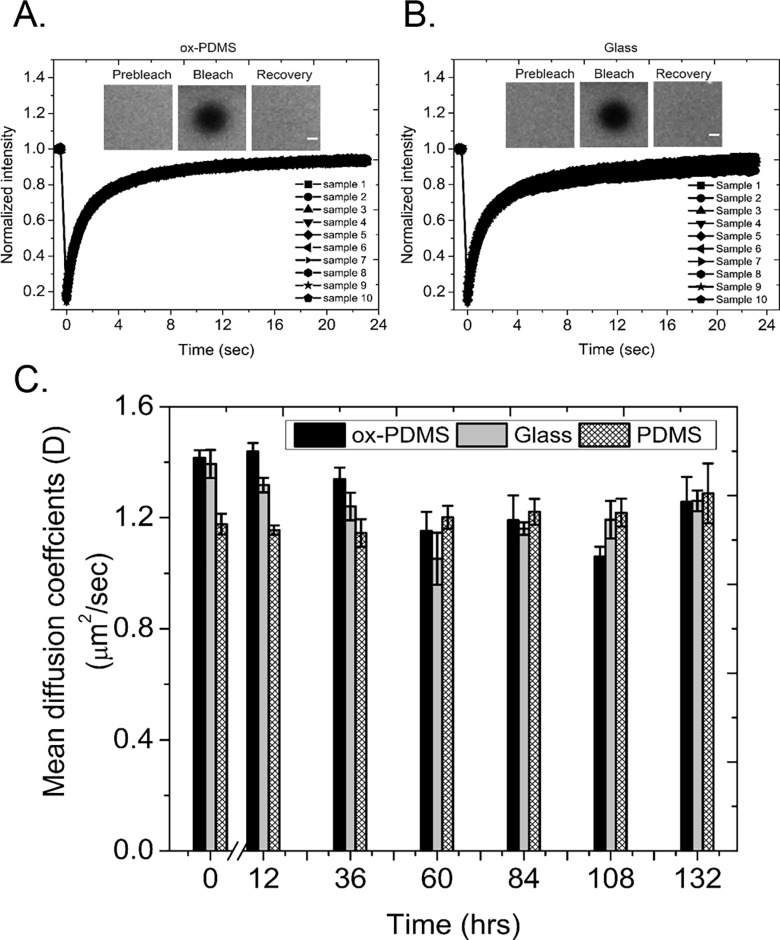
Measurement of the diffusive mobility of lipids on the lipid layers. Plot of the fluorescence recovery curves on, A) ox-PDMS, and B) glass, immediately after preparation of the lipid bilayers. Each plot shows recovery curves from FRAP measurements on ten different locations. The recovery curves are highly reproducible. Insets are representative images before the bleach pulse, the first image after the bleach pulse, and the final image of the time series at 24 seconds. Scale bars 2 μm. C) Plot of the diffusion coefficient versus the time since preparation of the lipid layer. The black bars are for the bilayers on ox-PDMS, the gray bars are for bilayers on glass, and the cross-hatched bars are for monolayers on native PDMS. The diffusion coefficient does not change significantly for the lipid monolayers over the course of 132 hours. The diffusion coefficient of the lipids in the bilayer on ox-PDMS and glass were similar and higher than the diffusion coefficient of the lipids in monolayers on native PDMS for the first 36 hours. The diffusion coefficient however, dropped by 20 percent at 60 hours and then matched those of the lipid monolayers prepared on PDMS. Each bar is a mean of *N* = 10 measurements. The error bars are standard deviations of the mean.

On ox-PDMS, PDMS, and glass, the fluorescence intensity of 5-μm diameter circular spots recovered rapidly after photobleaching. The recovery in intensity demonstrated that the phospholipids formed a continuous layer with free diffusive exchange of phospholipids within the plane of the membrane (**Fig 6A and 6B**). **Fig 6C** shows the diffusion coefficient calculated from the recovery curves. Each data point is an average of ten FRAP experiments. We find that on native PDMS the phospholipid monolayer exhibited *D* = 1.18 ± 0.04 μm^2^/s immediately after preparation. *D* did not change significantly over the course of 36 hours. At 60 hours after formation, we measured an increase in *D* of about ~ 4% to 1.20 ± 0.04 μm^2^/s. *D* remained in this elevated range for the remainder of our experiments. The diffusion coefficient measured on the phospholipid bilayer on ox-PDMS, *D* = 1.42 ± 0.03 μm^2^/s immediately after formation, was similar to that of bilayers on glass, *D* = 1.39 ± 0.05 μm^2^/s. For the first 36 hours, the diffusion coefficient of the bilayers on glass and ox-PDMS was higher than the diffusion coefficient of lipids on monolayers on native PDMS. This observation was consistent with those reported in the literature—phospholipids in monolayers appear to diffuse more slowly than phospholipids in bilayers. By one measure[33], fluorescent probes in DOPC phospholipid bilayers had a diffusion coefficient, *D* = 1.7 μm^2^/s on ox-PDMS, whereas similar probes in phospholipids monolayers on native PDMS had a diffusion coefficient, *D* = 0.9 μm^2^/s. The absence of a thin water layer that separates (and lubricates) the phospholipid headgroups from the solid support was attributed to the lowered mobility of the phospholipids in solid-supported monolayers in comparison to phospholipid bilayers[33].

After 36 hours, the diffusion coefficient of lipids on both the glass and ox-PDMS decreased by approximately 20 percent and became indistinguishable from that of the monolayer. Since lipid bilayers are reported to be stable indefinitely on glass[13,31], it is possible that the FRAP experiments are detecting changes in the lipids over time. One possibility is that the unsaturated double bonds in the alkylchains of DOPC was oxidizing due to prolonged exposure to an aqueous environment[35]. Increased saturation of the alkyl chain of lipids is known to increase the viscosity of lipid layers[34,36]. A higher viscosity causes a decrease in the apparent diffusion coefficient of the phospholipids[34,36]. Such a decrease would thus be independent of the characteristics of the substrate or the number of leaflets present in the lipid layer. We can conclude however, that the diffusive behavior of lipids in bilayers on ox-PDMS is indistinguishable from the diffusive behavior of lipids in bilayers on glass surfaces.

### The cleaning procedure significantly improved the quality of the bilayer on ox-PDMS

**Fig 7:**
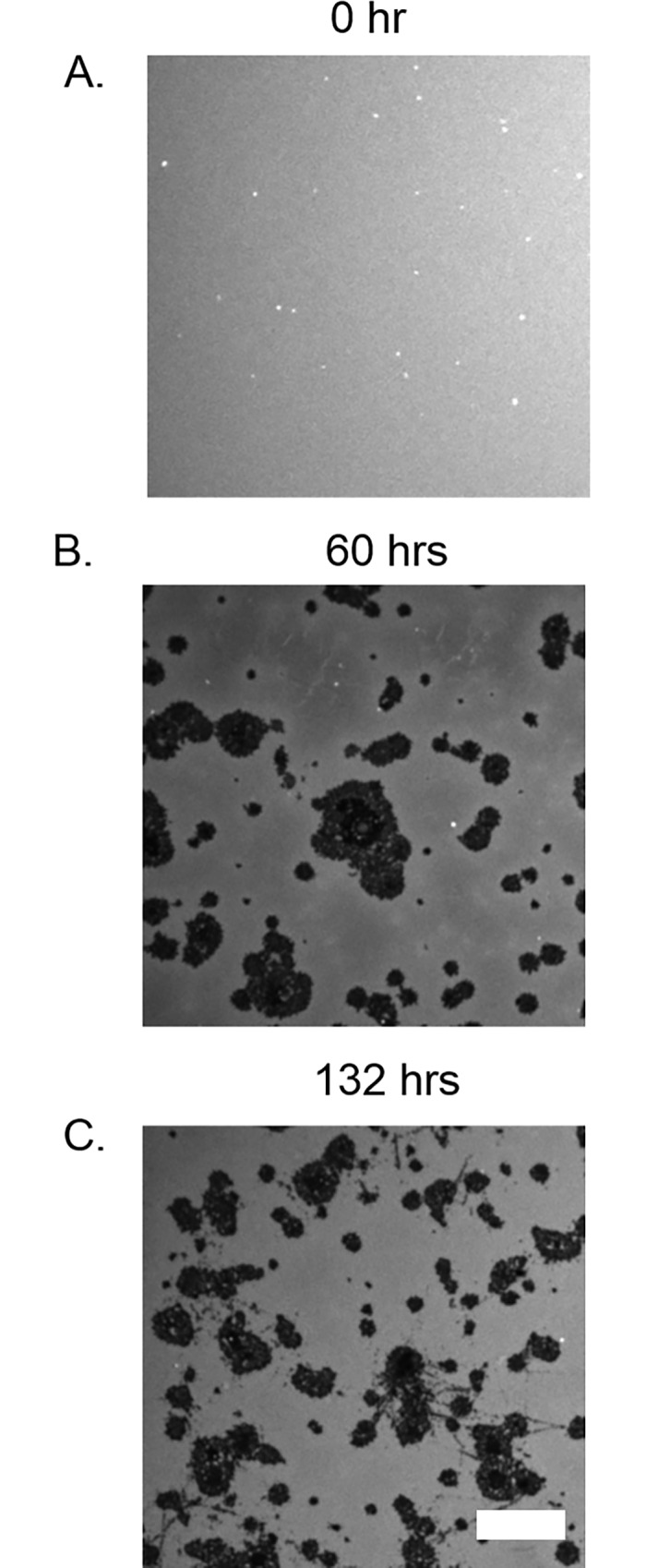
Confocal fluorescence images of the evolution of lipid bilayers on ox-PDMS not subjected to solvent sonication. A) The bilayer looks uniformly fluorescent immediately after the fusion of SUVs. B) At 60 hours, large areas of lipids seemed to have peeled away, revealing the non-fluorescent PDMS substrate (black patches). C) The dark regions became more extensive at 132 hours. Scale bar 10 μm.

We wished to determine if the cleaning procedure we employed had a discernible effect on the stability of phospholipid bilayers on ox-PDMS. **Fig 7** shows representative images, at 0 hours, 60 hours and 132 hours after preparation of phospholipid layers on ox-PDMS that was not subjected to the cleaning procedure. Similar to ox-PDMS that was solvent cleaned, a uniformly fluorescent phospholipid bilayer formed on the surface. After 36 hours however, large dark regions with no fluorescence appeared. These dark regions grew in size over the course of 132 hours. The dark regions are consistent with the phospholipid layer peeling off the surface of the PDMS. We conclude that solvent cleaning was essential to preserve the stability of lipid bilayers on ox-PDMS surfaces. It is known that unreacted monomers and oligomers that are present in the PDMS can diffuse through bulk PDMS and form a layer on ox-PDMS. Such a migration is favored thermodynamically in air and other non-polar environments since the hydrophobic monomers have a lower surface energy than the oxidized PDMS surface[23–28]. These monomers and oligomers contribute to the hydrophobic recovery of PDMS. We speculate that the unreacted monomers in the bulk, which were not removed since the PDMS was not sonicated in acetone and isopropanol, displaced the phospholipid molecules from the surface of PDMS. Since the bilayers supported on non-solvent cleaned ox-PDMS were of quality not suitable for most applications, we did not undertake a statistical study of growth of the defects or the diffusive characteristics of the lipids on these surfaces.

### Conclusions

Phospholipid bilayers on ox-PDMS were stable up to 132 hours after preparation, which makes ox-PDMS a suitable support for the majority of biophysical and single-use biosensor experiments involving phospholipid bilayers. The appearance of regions of higher fluorescence intensity after 36 hours was still consistent with these regions being bilayers. Cleaning of the PDMS with a three-step sonication procedure with acetone, isopropanol, and water proved essential for prolonging the stability of bilayers on ox-PDMS, and should be standard protocol for work with phospholipids on PDMS surfaces. Without this cleaning procedure, large patches of phospholipids came off the surface to reveal dark patches of bare PDMS.

## Supporting Information

S1 Fig Raw data demonstrating the presence of gradients in intensity in the majority of images. The plots in column A) are three-dimensional representations of raw pixel intensities from five confocal images of a lipid bilayer on ox-PDMS. The plots in column B) are three-dimensional representations of raw pixel intensities from five confocal image of a lipid monolayer on native PDMS.
(TIF)

S2 Fig Histogram of pixel intensities of lipid bilayers on ox-PDMS and lipid monolayers on PDMS from raw pixel intensities without correction of the gradients in intensity. The pixel distributions are broader for both monolayers and bilayers. The distribution for the bilayer appears to have a secondary peak. The mean intensity of the bilayer (the primary peak) is approximately double of the mean intensity of the monolayer.
(TIF)

S3 Fig Histogram of pixel intensities of lipid bilayers on glass. The imaging conditions for glass was different from those for the PDMS substrates resulting in shifted values for the raw intensities (555 nm diode laser was set at 1.0% power. The PMT gain was set to 700 whereas the PMT gain was set to 800 for imaging of PDMS and ox-PDMS). The pixel intensities are normally distributed however.
(TIF)
