# Supplementary figures and images for: Lipid Bilayers Are Long-Lived on Solvent Cleaned Plasma-Oxidized poly(dimethyl)siloxane (ox-PDMS)
Source: PLoS One. 2017 Jan 4;12(1):e0169487. doi: 10.1371/journal.pone.0169487 (PMC5214066; doi:10.1371/journal.pone.0169487)

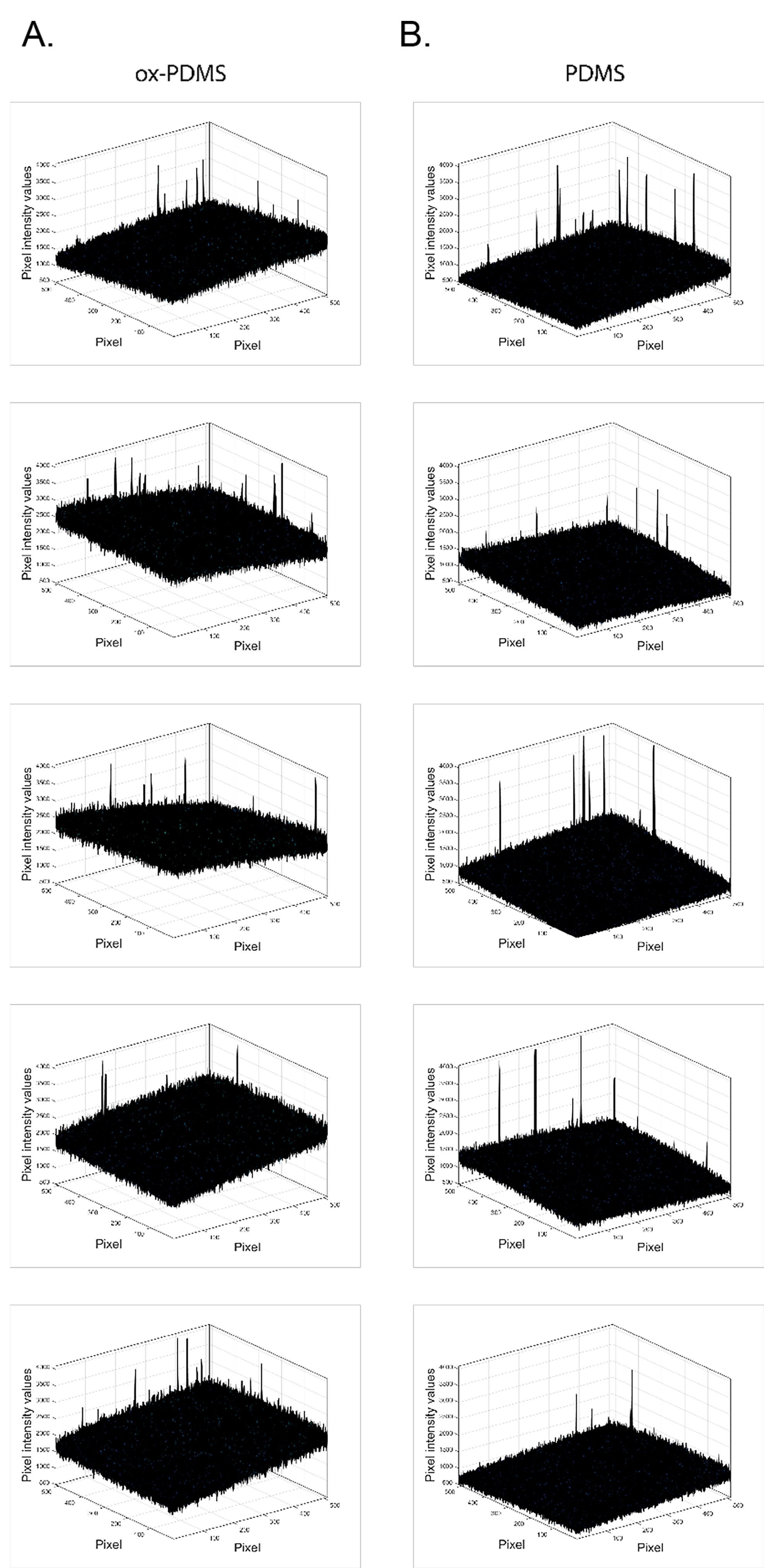

Supplement: S1 Fig — The plots in column A) are three-dimensional representations of raw pixel intensities from five confocal images of a lipid bilayer on ox-PDMS. The plots in column B) are three-dimensional representations of raw pixel intensities from five confocal image of a lipid monolayer on native PDMS. (TIF) [file pone.0169487.s001.tif]

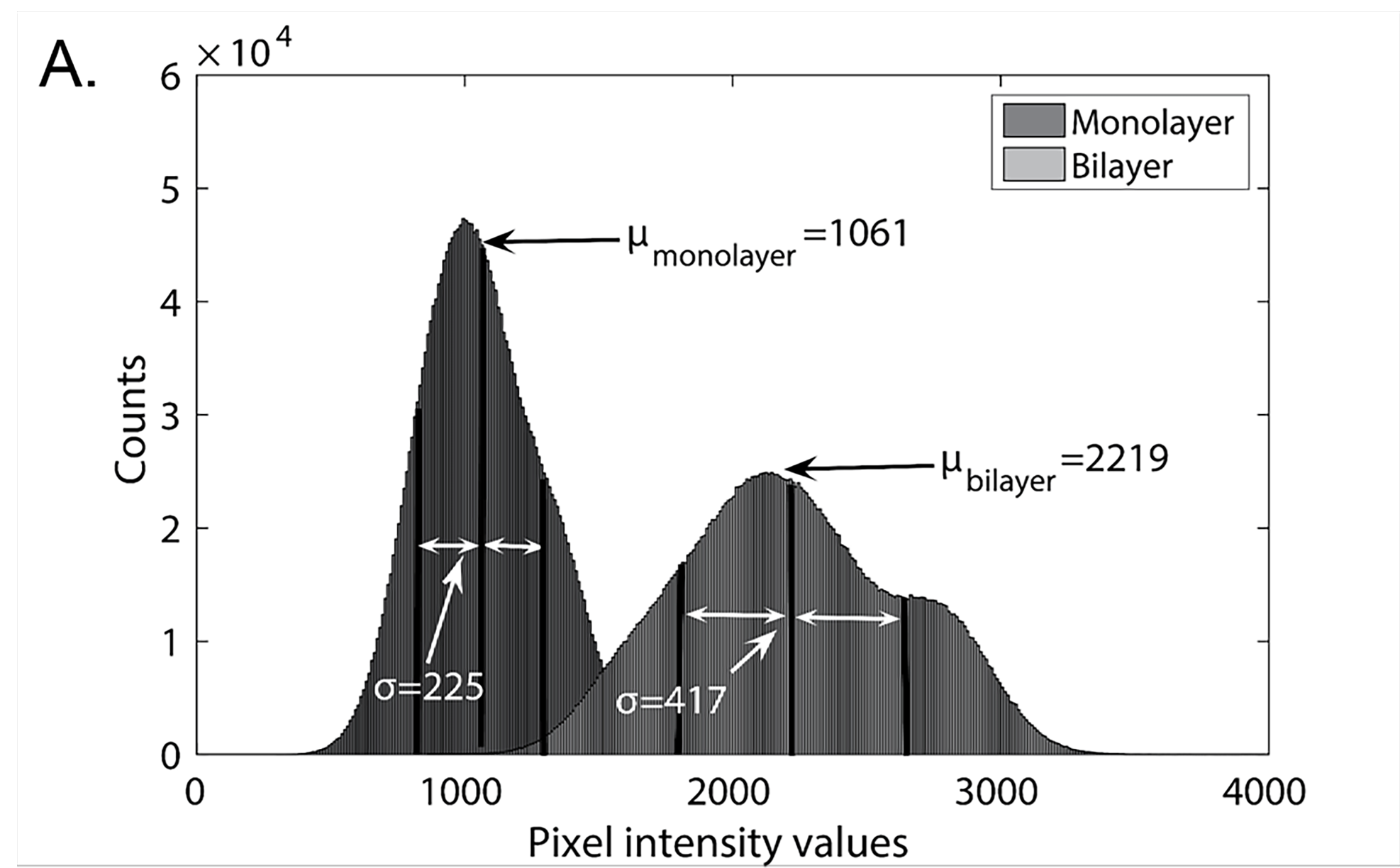

Supplement: S2 Fig — The pixel distributions are broader for both monolayers and bilayers. The distribution for the bilayer appears to have a secondary peak. The mean intensity of the bilayer (the primary peak) is approximately double of the mean intensity of the monolayer. (TIF) [file pone.0169487.s002.tif]

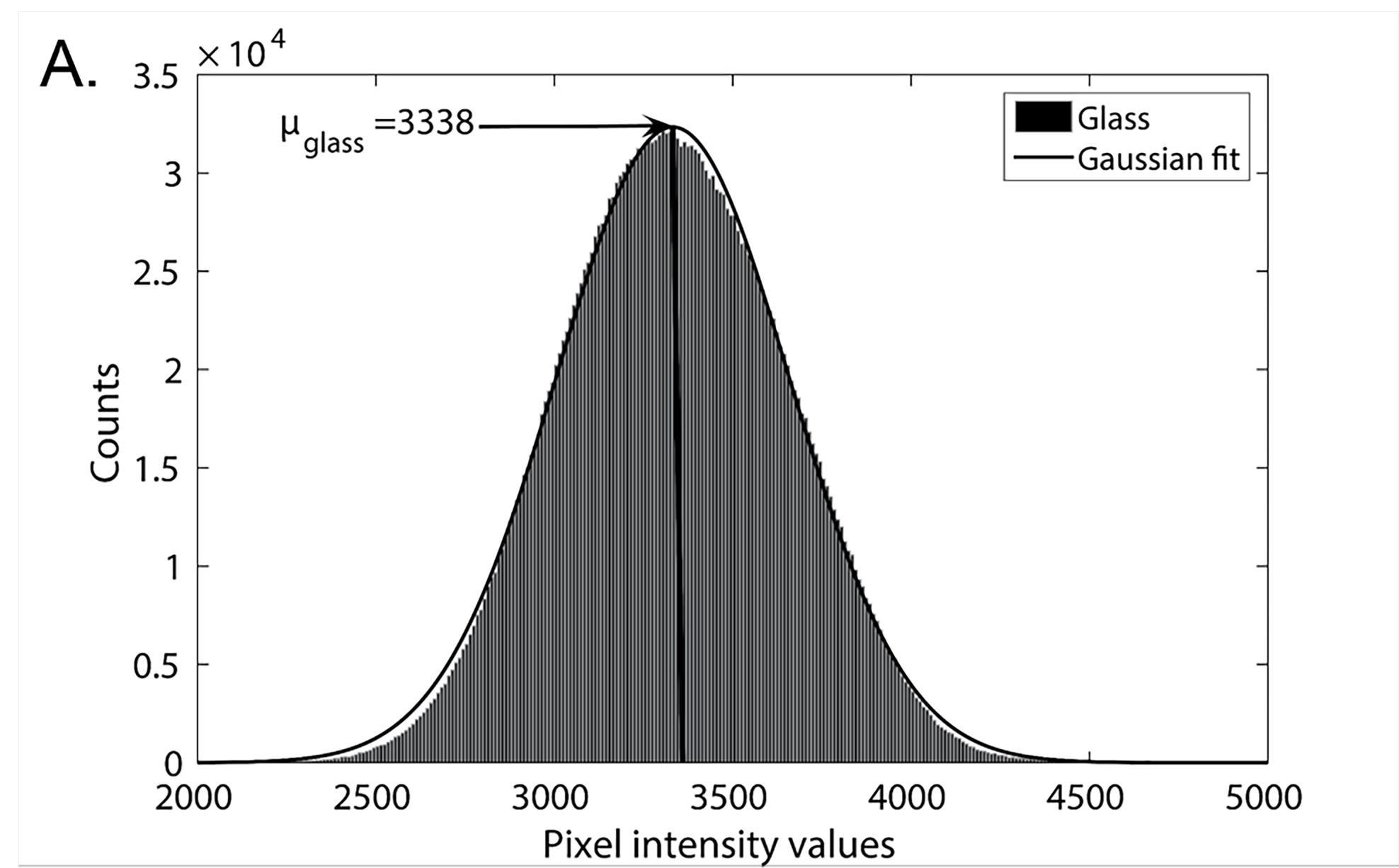

Supplement: S3 Fig — The imaging conditions for glass was different from those for the PDMS substrates resulting in shifted values for the raw intensities (555 nm diode laser was set at 1.0% power. The PMT gain was set to 700 whereas the PMT gain was set to 800 for imaging of PDMS and ox-PDMS). The pixel intensities are normally distributed however. (TIF) [file pone.0169487.s003.tif]
